# Contrast-Enhanced Ultrasound for Monitoring Treatment Response in Different Stages of Hepatocellular Carcinoma

**DOI:** 10.3390/cancers14030481

**Published:** 2022-01-18

**Authors:** Mariella Faccia, Matteo Garcovich, Maria Elena Ainora, Laura Riccardi, Maurizio Pompili, Antonio Gasbarrini, Maria Assunta Zocco

**Affiliations:** 1 Department of Internal Medicine, SS Annunziata Hospital Sulmona, 67039 Sulmona, Italy; mfaccia@asl1abruzzo.it; 2 Department of Internal Medicine and Gastroenterology, Fondazione Policlinico Universitario Agostino Gemelli IRCCS, Catholic University of Rome, 00168 Rome, Italy; matteo.garcovich@policlinicogemelli.it (M.G.); mariaelena.ainora@policlinicogemelli.it (M.E.A.); laura.riccardi@policlinicogemelli.it (L.R.); maurizio.pompili@policlinicogemelli.it (M.P.); antonio.gasbarrini@policlinicogemelli.it (A.G.)

**Keywords:** dynamic contrast-enhanced ultrasound, contrast-enhanced ultrasound, hepatocellular carcinoma, tumor response

## Abstract

**Simple Summary:**

The evaluation of tumor response to anti-cancer therapy is critical in oncology for the prompt determination of subsequent treatment and follow-up strategies. Historically, response criteria have been based on tumor size changes; however, since the development of locoregional and molecular-targeted therapies in HCC (which act by disrupting tumor vascularization rather than tumor cells), changes in tumor vascularity and enhancement patterns have been considered to be more reliable. Contrast-enhanced ultrasound (CEUS) and dynamic CEUS, which allow microvessel perfusion studies, are emerging as promising tools for early tumor response evaluation. This article provides a general review of the current literature regarding the usefulness of CEUS in monitoring HCC response to therapy, highlighting the role of the procedure at different stages of the disease.

**Abstract:**

The capacity of contrast-enhanced ultrasound (CEUS) to detect microvessel perfusion has received much attention in cancer imaging since it can be used to evaluate the enhancement patterns of the lesions during all vascular phases in real time, with higher temporal resolution as compared other imaging modalities. A rich body of literature has demonstrated the potential usefulness of CEUS in the assessment of HCC in response to both locoregional and systemic therapies. It is useful to evaluate the efficacy of ablation immediately after treatment to provide guidance for the retreatment of residual unablated tumors. In patients treated with transarterial chemoembolization (TACE), CEUS showed a high degree of concordance with computed tomography and magnetic resonance for the differentiation of responders from non-responders. Dynamic CEUS (D-CEUS) has emerged as a promising tool for the depicting changes in tumor perfusion during anti-angiogenetic treatment that can be associated with tumor response and clinical outcome. This article provides a general review of the current literature regarding the usefulness of CEUS in monitoring HCC response to therapy, highlighting the role of the procedure in different stages of the disease.

## 1. Hepatocellular Carcinoma

According to GLOBOCAN 2020, liver cancer is the sixth most diagnosed cancer and is the third leading cause of cancer-related death worldwide, with approximately 906,000 new cases and 830,000 deaths each year. Rates of both incidence and mortality are 2–3 times higher among men than among women in most regions, where liver cancer ranks fifth in terms of global incidence and second in terms of mortality for men [1].

Hepatocellular carcinoma (HCC) accounts for almost 90% of liver cancers and represents a major health challenge since its incidence and mortality are increasing in different parts of Europe and the United States despite prevention and surveillance strategies [2].

Improvements in HCC treatments are of paramount importance to achieving a substantial reduction in disease burden and increasing curative chances. In recent years, new anti-HCC treatments have progressively become more available. Their selection relies on tumor characteristics, the severity of underlying liver dysfunction, health status, local expertise, and medical resources. Since angiogenesis plays a fundamental role in the progression of hepatocellular carcinoma, a better characterization of intratumoral neovascularization represents a subject area with huge potential to advance our understanding on HCC behavior. In this regard, some studies are focusing on immunohistochemical assays to quantify CD34, CD31, and CD105 expression to estimate microvascular density and patterns and distinguish between benign and malignant hepatic nodules, with promising but still preliminary results [3,4].

Hepatic resection and liver transplantation are the mainstay curative treatments in early-stage HCC cases, while local ablation with radiofrequency (RFA) remains the backbone of image-guided treatment for non-surgical early cases. In this setting, microwave ablation is also emerging as promising tool for local control and survival. Transarterial chemoembolization (TACE) has been the standard of care for intermediate-stage cancer over the past two decades, while transarterial radioembolization (TARE) is emerging as a promising tool even if it is not established as a primary standard of care by guidelines. Finally systemic therapies, including immune-checkpoint inhibitors (ICIs), tyrosine kinase inhibitors (TKIs), and monoclonal antibodies, have been shown to lead to a marked increase in overall survival and quality of life in patients with advanced-stage HCC [5,6].

The evaluation of tumor response to anti-cancer therapy is critical to determining subsequent treatment and follow-up strategies. Historically, response criteria were based on tumor size measurement by radiological imaging techniques, and a reduction in tumor size during treatment was found to be associated with therapeutic and clinical benefits. However, the development of molecular-targeted and locoregional therapies in HCC, which act by disrupting tumor vascularization rather than destroying tumor cells, has led to the introduction of different methods to evaluate treatment efficacy. For this purpose, both Response Evaluation Criteria in Solid Tumors 1.1 (RECIST 1.1), which is based on the measurement of the whole tumor after treatment, and mRECIST, which considers the largest diameter of the residual arterialized tumor area, are recommended by the EASL guidelines [7,8].

Although contrast-enhanced computed tomography (CECT) and magnetic resonance imaging (CEMRI) are considered as the standard modalities for evaluating treatment efficacy, contrast-enhanced ultrasound (CEUS) represents a safe and cost-effective alternative for treatment outcome monitoring, given its ability to detect real-time microvessel perfusion during all vascular phases (arterial, portal venous, late, and post-vascular phases), with the advantages of higher temporal resolution and repeated examinations in a short time. Contrast agents consist of microbubbles made of a high-molecular-weight gas core surrounded by a lipid or albumin shell, with a size between 1 and 4 μm (smaller than erythrocytes), allowing an easy flow through small capillaries. Such agents, which are pure intravascular contrast mediums, enhance the backscatter of the ultrasound waves by resonance within sonic windows when administered into the vasculature, resulting in a marked amplification of blood flow signals and more detailed tissue perfusion studies as compared to CECT and CEMRI. This ultrasound-based tool provides not only a morphological data response based on mRECIST criteria, but also a functional data response through new software for the quantitative analysis of perfusion parameters that generate time–intensity curves of enhancement in selected regions (dynamic-CEUS, D-CEUS). This has led the European Federation of Societies for Ultrasound in Medicine and Biology (EFSUMB) to highlight the role of CEUS not only in the characterization and detection of focal liver lesions but also in monitoring tumor response after loco-regional and systemic HCC treatments [9,10]. However, CEUS can only be efficiently applied in patients who can be optimally scanned by gray-scale ultrasound. Major limitations are related to operator skills, acoustic windows in patients with obesity and meteorism, tumor location (lesions covered by the lung or diaphragm), and multiplicity, which requires additional doses of contrast agent to ensure that no lesions are missed and to clarify the characteristics of each lesion. Such drawbacks may reduce CEUS reproducibility.

This article provides a general review of the current literature regarding the usefulness of CEUS and D-CEUS in monitoring HCC response to different treatment strategies. For this purpose, we conducted a literature review through a systematic and comprehensive database search (PubMed, Cochrane Library, Scopus, Web of Science), using the following terms: “contrast-enhanced ultrasound”, “hepatocellular carcinoma” and “treatment response” in July 2021. The reference lists of the included articles were hand-searched to identify additional studies of interest.

## 2. Technical Considerations Regarding Dynamic CEUS Application

The introduction of CEUS has improved the detection and characterization of focal lesions in comparison to conventional ultrasounds. Moreover, in recent years new ultrasound equipment and analysis software has enabled the application of D-CEUS imaging. This ultrasound feature allows us to objectively quantify tissue and tumor enhancement to evaluate therapeutic response after HCC treatment.

**Figure 1:**
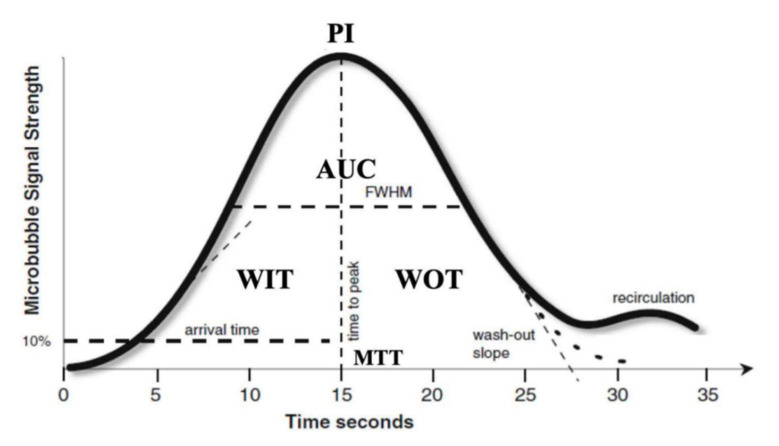
An example of a time–intensity curve after CEUS examination and an explanation of the most important parameters. Microbubble signal strength is usually measured using absolute intensity units and time in seconds. Abbreviations: time to peak, TP; wash-in time, WIT; wash-out time, WOT; peak intensity, PI; mean transit time, MTT; area under the curve, AUC.

D-CEUS is usually performed through digitized quantification of contrast uptake on a recorded video clip of a standard CEUS examination by means of analysis software embedded directly in an ultrasound device or exported on external computers. These “plug-ins” are tools that generate time/intensity data by analyzing the image pixel content, resulting in time–intensity curves (Figure 1) which provide an estimation of parameters related to tissue/tumor perfusion. Time–intensity curves are drawn from a region of interest (ROI) that is placed over a target lesion large enough to encompass the whole tumor surface, while the subsequent analysis displays the mean, median, and standard deviation of intensity pixel within the ROI for each image frame of the CEUS examination.

Some perfusion parameters that characterize both blood volume and blood flow and that can be found in most of the experimental studies involving D-CEUS as follows:

‒ Time to peak (TP) is the time from zero intensity (immediately before the contrast arrives in the ROI) to maximum intensity.
‒ Wash-in time (WIT) is time from 5% intensity to 95% intensity.
‒ Wash-out time (WOT) is the time from the peak of the curve to the zero value again.
‒ Peak intensity (PI) is the maximum intensity on the time–intensity curve.
‒ Mean transit time (MTT) describes the mean time taken by the CEUS bubbles (from the time of first arrival in the ROI) to pass through the ROI. Mathematically it is the first moment of the TIC curve. This parameter is easily calculated from the fitted mathematical model and is often given in a closed-form analytical expression.
‒ Area under the curve (AUC) is the area under the time–intensity curve above baseline and is calculated numerically between the starting time and a predefined end time. This parameter may be related to blood volume.

## 3. Radiofrequency Ablation and CEUS

RFA is regarded as one of the best treatment options for patients with early-stage HCC who are not suitable for surgical resection or liver transplantation. It acts by causing heat-induced coagulative necrosis of the target lesion through an applicator directly inserted into the tumor under imaging guidance [6]. The curative effect of RFA and long-term survival of the patient are strongly dependent on tumor size and number, serum tumor markers, proximity to a large vessel, insufficient safety margins (<5 mm), subcapsular location, and poor pathologic differentiation of tumor cells. Complete tumor necrosis is almost 90% for HCCs < 2 cm and drops to 50–70% for lesions between 3 and 5 cm due to the heat loss from perfusion-mediated tissue cooling within the ablated region [6,11]. However, a potential extension of RFA application derives from the promising results of a recent retrospective study showing multiprobe stereotactic RFA with intraprocedural fusion imaging as an effective method for the treatment of HCC lesions exceeding 3 cm. This study reported a complete response rate in 96.2% of cases in patients undergoing bridging therapy, using histological analysis of HCC transplanted livers as a reference [12].

Therefore, early diagnosis of local recurrence and timely retreatment are strategic for optimizing HCC management. CECT performed 4 weeks after treatment is nowadays considered the diagnostic gold standard; however, a growing number of studies have investigated CEUS performance in this setting. Attractive results were obtained by Ricci et al. and Wang et al., who reported the sensitivity, specificity, negative predictive value (NPV), and positive predictive value (PPV) of CEUS for identifying residual unablated tumors as compared to CECT, obtaining values of 92%, 100%, 97%, and 100%, and 88%, 99%, 94%, and 97%, respectively [13,14]. CEUS showed poorer performance in the study by Zheng et al., showing sensitivity, specificity, PPV, NPV, and overall accuracy values of 68%, 97%, 82%, 94%, and 92% in detecting local tumor progression, and 78%, 92%, 92%, 77% and 84%, respectively, in detecting new intrahepatic recurrent foci, with respect to CECT [15].

**Figure 2:**
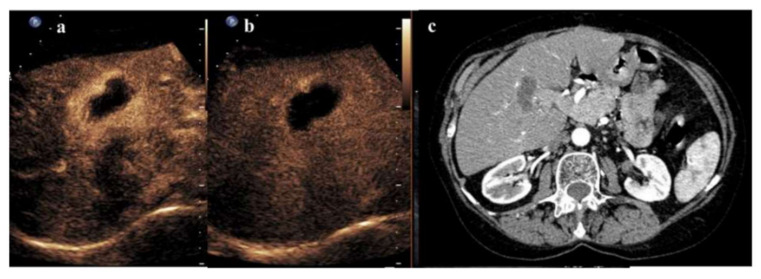
A 2.2 cm solitary HCC in segment IV with complete response after RFA. (**a**) Arterial phase CEUS (8 s after SonoVue injection) shows an avascular pattern with peripheral rim enhancement. (**b**) Late-phase CEUS (141 s after SonoVue injection) confirms an avascular pattern with persistent peripheral rim enhancement, suggesting reactive hyperemia. (**c**) Arterial phase CECT 1 month after RFA confirms complete necrosis.

In these studies, false negatives reflected the known drawbacks of CEUS, in particular the difficulties in evaluating deeply located HCCs (such as posterior and subphrenic lesions) and including large lesions in the observing acoustic window; the impossibility of detecting the entire liver parenchyma during the arterial hyper-enhancement scan in order to identify the appearance of new foci; and the low image quality in the presence of lung and intestine gas. False positives were mainly linked to the persistence of hyperemic halos around ablated tissue because of inflammatory reactions to thermal injury, limiting the use of all imaging modalities early after the procedure. A pilot study on post-procedural CEUS, performed within 1 h of ablation, showed a sensitivity of 40% and a specificity of 94% as compared to follow-up CECT or CEMRI [16]. In a study by Meloni et al., sensitivity and specificity values in the early evaluation of liver tumors following RFA were 25% and 96% for immediate CEUS, 20% and 97% for CEUS at 24 h, and 40% and 97% for CECT at 24 h, as compared to 3-month imaging follow-up. Researchers identified some useful characteristics for differentiating the peripheral rim halo from residual tumor tissue since the former usually appears to be uniform in thickness, shows later arterial enhancement, and does not display portal and late-phase wash-out [17]. However, in a study by our group including 104 unresectable HCCs, CEUS preformed 48 h after locoregional therapies showed sensitivity, specificity, PPV, NPV, and accuracy of 79%, 97%, 94%, 87%, and 89%, respectively, in detecting treatment failure with respect to CECT at 1 month. According to our results, CEUS performed 48 h after treatment can be considered a reliable modality for the early evaluation of treatment efficacy; however, more data are warranted to strengthen these conclusions [18]. An example of a single HCC nodule treated by RFA and evaluated by CEUS after 48 h is shown in Figure 2.

Other studies have investigated perfusion patterns of recurrent HCCs after RFA to identify typical behavior using CEUS with respect to the initial lesion. Leoni et al. reported isoenhancement in all vascular phases in >50% of recurrent lesions, while Wu et al. observed that recurrent HCCs had more homogeneous enhancement in the arterial phase, poorly defined borders at the peak, marked wash-out, fewer feeding vessels, and more inner necrotic areas as compared to initial HCCs because of blood sinus expansion surrounding the ablation area induced by thermal ablation. The further definition of perfusion features in local recurrence of HCC would help to optimize treatment strategies [19,20].

D-CEUS applications in this field are limited but promising since it enables a quantitative assessment of tumor perfusion, which is known to influence energy distribution of RFA. Some studies reported that a low peak intensity (PI) of the lesion before RFA treatment was a significant risk factor for intrahepatic recurrence, hypothesizing a thermal sink effect leading to incomplete ablation secondary to rapid blood outflow from a drainage route [21,22,23]. The predictive value of low time to peak (TTP) values before RFA is controversial [21,22].

It is worth mentioning that CEUS also plays an important role during the percutaneous treatment procedure, guiding the correct positioning of the needle inside the lesion. According to Solbiati et al., the routine adoption of CEUS in the ablative procedure decreased the rate of partially treated tumors detected using CECT within 1 week after treatment from 16.1% to 5.9% in a group of 429 HCC and metastatic lesions [24].

## 4. TACE and CEUS

TACE is the recommended first-line therapy for patients with intermediate-stage HCCs, particularly for those with pauci-nodular disease without vascular invasion or metastases, who are asymptomatic, and have a Child-Pugh stage of ≤B7. In this case the survival is increased up to 40–50 months after treatment. TACE is also used as a bridging therapy to downstage the disease in transplant-eligible patients. It consists of the injection of a chemotherapeutic drug followed by an embolic agent into the tumor-feeding branch of the hepatic artery to create an ischemic environment and trap the chemotherapy within the tumor. Conventional TACE (C-TACE) uses an emulsion of chemotherapy mixed with iodized oil (lipiodol) followed by gelfoam particles or other embolization materials, while the more recent drug-eluting bead TACE (DEB-TACE) uses polyvinyl alcohol-based microspheres loaded with anthracycline drugs such as doxorubicin [6,25].

Imaging follow-up studies consist of CECT and/or a CEMRI performed 4 to 6 weeks after therapy. This time frame has been established based on the need to differentiate peripheral viable tumors from inflammatory peritumoral infiltration and according to the experience with CECT for C-TACE control, since the non-tumoral liver parenchyma requires 3–4 weeks to eliminate lipiodol by Kupffer cell phagocytosis, causing overestimation of TACE tumor control by CECT [26,27]. To overcome this limitation, CEUS has been investigated as an earlier follow-up imaging tool for TACE, with attractive results. Takizawa et al. reported that the detection rate of residual HCC lesions was significantly higher with CEUS performed after 1 day than with CECT performed after 1 month (95.7% vs. 78.7%, *p* < 0.05); similarly, Watanabe et al. showed that CEUS 1 to 2 days after TACE was not inferior to CECT at 4 weeks after TACE, with the advantage of allowing early retreatment [28,29]. In a study by Xia et al., the detection rates of positive enhancement with CEUS and CECT after 1 week were 58.1% and 39.5%, respectively, confirming that CEUS at 1 week post-treatment was statistically more sensitive (*p* < 0.01) than CECT in depicting residual blood supply of HCCs shortly after TACE [30]. In another study, the sensitivity and accuracy values for detecting residual tumor by CEUS and CECT, performed contemporaneously within 0.5 to 3 months after TACE (maximum 2 weeks apart), were 95.9% vs. 76.2% (*p* < 0.001) and 96.2% vs. 77.7% (*p* < 0.001), respectively, using histology or angiography as a reference standard. In this study both CEUS and CECT missed new lesions (3 vs. 2) which were detected by the other imaging modality, suggesting that a combined approach may be superior to either imaging approach alone [31].

Similarly, Shaw et al. found that CEUS assessment of residual blood flow at 1–2 weeks and 1 month after treatment with DEB-TACE was comparable to that of CECT and CEMRI, and that CEUS was particularly useful in the first 1–2 weeks after treatment [32]. In the study by Shiozawa et al., CEUS at 3 days after DEB-TACE allowed the early assessment of therapeutic efficacy, with no enhancement and peripheral ring enhancement significantly suggestive of a positive outcome [33]. Moschouris et al. reported the utility of CEUS in increasing the sensitivity, specificity, and diagnostic accuracy of CECT after DEB-TACE (80.4%, 88.9%, and 81.8% for CECT and 93.5%, 100%, and 94.5% for CEUS + CECT, respectively) using CEMRI as reference standard [34].

**Figure 3:**
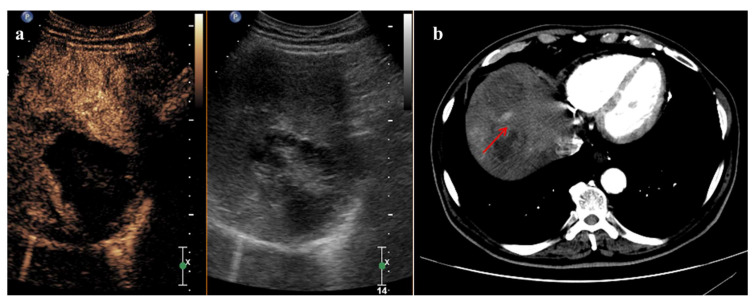
A 5 cm HCC of the segment VIII with discordant behavior after combined therapy (RFA + TACE). (**a**) Arterial phase imaging (22 s after SonoVue injection) shows no enhancement within the lesion. (**b**) Arterial phase CECT scan 1 month after treatment shows the persistence of viable tumor (red arrow) in the upper posterior part of the lesion not detected by CEUS.

Multi-nodularity, hypoenhancement in pretreatment CEUS and infiltrative diffuse growth patterns are tumor conditions that affect CEUS efficacy. CEUS provided an accurate assessment of treatment response to locoregional therapies using 1 contrast injection in 85% of solitary tumors, 58% of 2 treated tumors and 67% of 3 treated tumors, respectively [35]. An example of a discordant behavior after combined therapy (TACE + RFA) between CEUS and CECT is shown in Figure 3.

The role of D-CEUS in post-TACE success control has been investigated in a limited number of studies and no consensus has been reached. Moschouris et al. reported a statistically significant decrease in perfusion index after DEB-TACE; similar figures were obtained by Nam et al., showing that PI values with 3D CEUS at 1–2 weeks and with 3D/2D CEUS at 1 month were significantly lower in the complete treatment group than in the incomplete group [36,37]. Furthermore, Rennert et al. showed a significant reduction in peak enhancement between the center of the lesion, its border areas, and the surrounding liver, with no significant differences concerning other parameters such as the wash-in area under the curve (WiAuC), mean transit time (MTT), and TTP [38]. On the contrary, in the study of Jiang et al. no significant changes in the rise time or peak time after TACE were observed [39]. Heterogeneity in number, size, and vascularization of the tumor could explain the disagreement.

Furthermore, a pilot study of 13 TACE-treated HCCs identified another potential use of CEUS, which showed an accuracy of 85% in identifying the feeding vessel in persistent viable HCCs after initial TACE using angiography as a reference standard [40].

Finally, it is worth mentioning that mRECIST-applied CEUS yielded not only diagnostic accuracy but also a high prognostic value after 1 month of the third DEB-TACE treatment for HCC in comparison to mRECIST evaluated in CECT/CEMRI. Responders (complete + partial response) according to mRECIST-CEUS had a significantly longer mean OS and time to progression compared to non-responders (37.1 vs. 11.0 months, *p* < 0.001 and 24.6 vs. 10.9 months, *p* = 0.007, respectively) [41].

## 5. Systemic Therapies and CEUS

Molecular targeted therapies with multi-kinase inhibitors (MKIs) are recommended for patients with preserved liver function and advanced-stage HCC as first and second line treatments [6]. These agents work by disrupting signaling pathways involved in angiogenesis, oncogenesis, and the tumor microenvironment and are usually well tolerated. However, some patients show severe adverse events and poor survival benefit; therefore, the early evaluation of therapeutic response to MKIs is of major importance as it permits the identification of non-responders to treatment, sparing unnecessary toxicity and costs.

Based on their unique mechanism of action, MKIs induce changes in tumor vascularity and structure prior to triggering changes in tumor size, making purely dimensional criteria unsuitable to define the response to treatment. For this purpose, D-CEUS is currently under evaluation for monitoring anticancer efficacy based on perfusion parameter changes, as reported in other solid tumors on antiangiogenic treatment [42,43].

**Figure 4:**
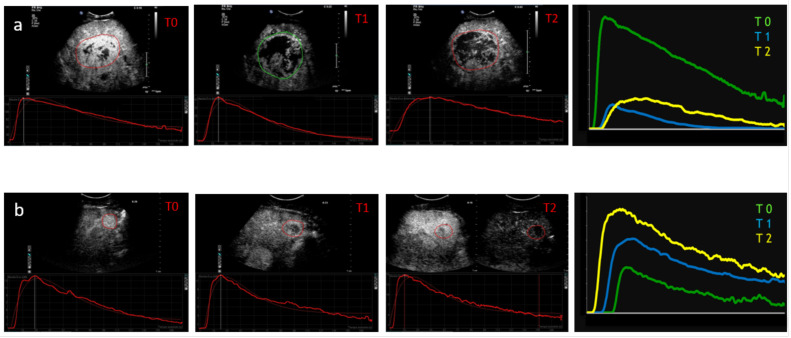
Two examples of HCC patients undergoing systemic therapy. (**a**) Contrast-enhanced ultrasound with the corresponding time–intensity curve at baseline (T0). At 15 (T1) and 30 days (T2) after the onset of sorafenib therapy, CEUS revealed an increase in tumor necrosis with a drastic reduction in tumor perfusion parameters, as shown by the contrast enhancement pattern and the corresponding time-intensity curve. Time–intensity curves of tumor enhancement are shown at baseline (green curve), on day 15 (blue curve), and on day 30 (yellow curve). It is possible to observe a reduced maximum enhancement and lower area under the enhancement curve early after treatment. Intensity is measured in absolute intensity units (AIU) on the ordinate, while time is measured on the abscissa (in seconds). (**b**) Contrast-enhanced ultrasound images and the corresponding time intensity curves of HCC lesion before and after treatment with sorafenib are shown in a non-responder patient. The CEUS and the corresponding time–intensity curves of tumor enhancement show no significant difference in perfusion parameters at baseline (T0, green curve), on day 15 (T1, blue curve), or on day 30 (T 2, yellow curve). Intensity is measured in absolute intensity units (AIU) on the ordinate, while time is measured on the abscissa (in seconds).

Sorafenib is the progenitor of MKI introduced for first-line treatment of advanced-stage HCC and leads to a median survival and a time to radiologic progression nearly 3 months longer as compared to placebo treatment. A cystic transformation often combined with tumor enlargement on CEUS was described in a group of 21 HCCs with no response to sorafenib treatment, but these findings do not seem accurate in the absence of contrast enhancement information about persistently viable areas, [44]. In the study of Frampas et al. on 11 patients treated with sorafenib and sunitinib, a decrease in the AUC of more than 40% at 1 month using D-CEUS predicted non-progression with respect to RECIST at 2 months evaluated using CECT [45]. The percentage changes of the AUC both at day 15 and at day 30 with respect to baseline were confirmed as predictive of response in the study of Lassau et al., which included 539 patients with different tumors including HCCs in 19 French centers [46]. In the study of Sugimoto et al. on 37 HCC patients treated with sorafenib and studied with D-CEUS with sonazoid as a contrast agent, AUC during wash-in on day 14 was the most relevant parameter for tumor response prediction, while AUC during wash-in on day 7 was the most relevant parameter for both PFS and OS compared with the results of CECT at 1 month [47]. In our study involving 28 HCCs, a significant correlation was reported between a reduction in 3 D-CEUS parameters (PI, AUC, and slope of wash-in (Pw)) and OS/PFS after 15–30 days of sorafenib therapy. Patients whose total blood volume (described by AUC) decreased by at least 10% after 2 weeks of treatment had a higher median overall survival (12.5 vs. 6.7 months) than those with an increase or a reduction of less than 10%. A trend toward correlation between the increase in time to PI (TP) and tumor response was also observed, although this association was not statistically significant [48]. Moreover, in another study TP at 1 month was the best discriminator for responders and non-responders (273 days versus 214 days) and this parameter continued to increase in the responder group because of the impact of antiangiogenetic treatments on dynamic flow [49]. However, it is worth mentioning that the presence of portal vein thrombosis influences hemodynamic parameters and TP results. Two examples of HCC patients undergoing systemic therapy (one responder and one non-responder) with sorafenib then evaluated by longitudinal D-CEUS imaging are shown in Figure 4.

Lenvatinib is another MKI introduced for the first-line treatment of advanced-stage HCCs. In a small study of 20 lenvatinib-treated HCCs, Kuorda et al. reported that the rate of change of Pw, TTP, and AUC on CEUS at day 0 and day 7 was significantly different in the responders and non-responders, reflecting the results of the CECT evaluation performed 8 weeks after starting therapy [50].

## 6. Conclusions

Early changes in tumor enhancement patterns and perfusion on CEUS may be predictive of tumor response, PFS, and OS in patients with HCC. Dynamic changes in tumor vascularity are detected earlier using D-CEUS as compared to the current reference standard imaging techniques.

Accurate early measurement of the effectiveness of locoregional and antiangiogenic agents would enable the rapid optimization of therapy in individual patients and spare unnecessary toxicity and costs. For long-term surveillance, CEUS and D-CEUS should be complemented with CECT/MRI for a comprehensive evaluation of the disease.
